# mRNA-seq-based analysis predicts: AEG-1 is a therapeutic target and immunotherapy biomarker for pan-cancer, including OSCC

**DOI:** 10.3389/fimmu.2024.1484226

**Published:** 2024-10-17

**Authors:** Lihong Yao, Lixue Liu, Wanqiu Xu, Hualei Xi, Song Lin, Guiyan Piao, Ying Liu, Jinrong Guo, Xiumei Wang

**Affiliations:** Department of Stomatology, The Second Affiliated Hospital of Harbin Medical University, Harbin, Heilongjiang, China

**Keywords:** AEG-1, pan-cancer, OSCC, immune infiltration, mRNA-seq

## Abstract

**Background:**

The aberrant expression of AEG-1 is significantly correlated with tumorigenesis, development, neurodegeneration and inflammation. However, the relationship between AEG-1 expression and immune infiltration in OSCC, as well as other tumor types, has yet to be comprehensively analyzed.

**Methods:**

The expression levels, prognostic and clinicopathological characteristics, mutation patterns and methylation landscapes of AEG-1 in various tumors were obtained from multiple databases, including TIMER, GEPIA, HPA, TCGA, UALCAN, cBioPortal, SMART and TISIDB, in addition to single-cell RNA-seq data. The integration of these datasets facilitated the elucidation of the relationships among pan-cancer cellular heterogeneity, immune infiltration and AEG-1 expression levels. *In vitro* experiments created AEG-1 overexpressing cell lines, and mRNA-seq analyzed AEG-1-related differential genes in OSCC. RT-PCR validated these findings *in vivo* using xenograft tumors. Tumor cell lines were developed to study AEG-1’s effects through H&E, Masson, and PAS staining. Immunohistochemistry examined AEG-1-related gene expression patterns.

**Results:**

Our analysis demonstrated that AEG-1 is highly expressed across various cancer types and is associated with tumor grade and patient prognosis. Additionally, AEG-1 amplification was observed in multiple cancers. Notably, we identified a significant elevation of AEG-1 expression in OSCC, which strongly correlated with patient prognosis and immune infiltration. Through mRNA-seq analysis of differentially expressed genes and immune-related gene sets, we identified a strong correlation between AEG-1 and immune infiltration markers such as LCP2, CD247, HLA-DPA1, HLA-DRA, HLA-DRB1, CIITA and CD74 in OSCC. Additionally, AEG-1 was found to regulate Th1/Th2 immune homeostasis, promote glycogen accumulation, and contribute to tumor fibrosis.

**Conclusion:**

In conclusion, AEG-1 significantly correlates with prognosis and immune infiltration across various cancer types and holds potential as a novel prognostic immune biomarker for OSCC. This finding may facilitate the identification of patients who are most likely to benefit from adjuvant immunotherapy.

## 1 Introduction

The immune system constitutes the organism’s primary defense mechanism against pathogenic invasion, tasked with the detection and elimination of foreign bodies and pathogenic microorganisms. Nonetheless, hyperactivity of the immune system can result in deleterious effects on the organism’s organs and tissues (1, 2). Furthermore, the immune system exhibits an antagonistic role in tumorigenesis (3).

Oral squamous cell carcinoma (OSCC) constitutes a significant subset of head and neck squamous cell carcinoma (HNSCC) and continues to be among the most fatal malignancies worldwide (4). The condition exhibits a high prevalence, mortality rate, and teratogenicity, collectively posing a substantial threat to human life and health (5). An increasing body of research has demonstrated that tumor development can be regulated by various genes, proteins, and molecular compounds, enabling tumor cells to evade immune responses, immune surveillance, and develop resistance to chemotherapy. Immunotherapy for tumors aims at modulating various immune processes and critical checkpoints, such as cytotoxic T-lymphocyte-associated antigen-4 (CTLA4) and the programmed death protein 1 (PD-1) along with its ligand (PD-L1) (6, 7). However, only a limited subset of patients derive clinical benefit from these treatments. In the context of clinical pharmacotherapy, the four-year survival outcomes associated with drug K (pembrolizumab) in patients with HNSCC signify a substantial advancement in the application of immunotherapy for recurrent or metastatic HNSCC. This breakthrough has garnered significant attention towards HNSCC within the broader scientific community and represents a noteworthy advancement in oncology. However, the overall survival rate for patients with OSCC has not shown substantial improvement, with the 5-year survival rate persisting below 50% (8, 9). Consequently, it is crucial to identify novel immunotherapeutic targets capable of overcoming tumor resistance and eliciting a robust immune response to eradicate tumor cells, thereby enhancing patient survival and quality of life.

The Astrocyte Elevated Gene-1 (AEG-1), also referred to as MTDH or LYRIC (10), is located on chromosome 8q22.20 (11) and is characterized by rapid subtraction hybridization (RaSH). AEG-1 was initially identified in primary human fetal astrocytes (PHFAs) through RaSH as an inducible gene for HIV-, gp120-, and tumor necrosis factor-α (TNF-α) (12–17). Its primary localization site has been identified as the endoplasmic reticulum (ER). AEG-1 is markedly overexpressed in various cancers, facilitating several oncogenic characteristics such as proliferation, invasion, metastasis, angiogenesis, and chemoresistance (18). Additionally, it is regulated by miRNAs (19). These pathways, including phosphatidylinositol-3-kinase/AKT (PI3K/AKT), NF-κB, mitogen-activated protein kinase (MAPK), and Wnt signaling, mediate the oncogenic function of AEG-1 and regulate angiogenesis and drug resistance. Nevertheless, the relationship between these factors and tumor immune cell infiltration remains to be fully elucidated.

In this context, we performed an analytical study utilizing public databases and mRNA-seq data to thoroughly investigate the role of AEG-1 in OSCC compared to other tumor types. Our research specifically involved mRNA expression profiling, prognostic value analysis, differential expression gene ontology analysis, and examination of correlations with tumor-infiltrating immune cells. This approach aimed to elucidate the relationship between AEG-1 expression, favorable overall survival in OSCC, and the positive modulation of the immune response in OSCC. These findings suggest that AEG-1 expression could function as a prognostic indicator and a critical biomarker for informing therapeutic strategies in patients with OSCC.

## 2 Materials and methods

### 2.1 AEG-1 gene expression analysis

The TIMER database (https://cistrome.shinyapps.io/timer/) (20)is an analytical network about the infiltration of tumor-infiltrating immune cells (21). In addition to evaluating the infiltration levels of tumor-infiltrating immune cells, the database also examines gene expression disparities between tumor tissues and normal tissues. Given that the TIMER database exclusively incorporates TCGA data and does not provide exhaustive coverage of specific tumor types, we performed a supplementary search utilizing both TCGA and GTEx data through the GEPIA database (http://gepia.cancer-pku.cn) (22) to identify tumors not represented in TIMER. To determine significant differences, we utilized the Wilcoxon test. Additionally, the expression level of the AEG-1 protein in HNSC was analyzed using data from HPA database (https://www.proteinatlas.org/) (23).

### 2.2 Prognostic and clinicopathological characterization of AEG-1

Clinical data for 33 tumor types were obtained from the TCGA database. Four survival outcomes were chosen for analysis: overall survival (OS) (24), disease-free survival (DFS) (25), disease-specific survival (DSS) (26), and progression-free survival (PFS) (24). The aim was to determine the association between AEG-1 expression and the prognosis of these 33 cancers. Cox regression analysis was employed, and the results were visualized using a forest plot. The UALCAN database (ualcan.path.uab.edu/analysis) (27) serves as a comprehensive, user-friendly, and interactive web resource for the analysis of cancer-related omics data, offering access to publicly available datasets such as TCGA, MET500, CPTAC, and CBTTC. This database investigated the correlation between AEG-1 expression and various clinical parameters, including age, gender, weight, and tumor grading, in patients diagnosed with HNSC.

### 2.3 Mutation analysis of AEG-1

The cBioPortal (https://www.cbioportal.org/) (28) is a web-based platform to analyze oncogenomic characterization of the AEG-1 gene. The database was employed to investigate the mutation frequency of AEG-1.

### 2.4 AEG-1 with TMB, MSI, and DNA methylation analysis

Tumor mutation burden (TMB) (29), defined as the total number of somatic cell-based coding errors, including base substitutions and insertion-based errors per million bases, is the most recent biomarker for evaluating the efficacy of PD-1 antibodies and their impact on tumors. Microsatellite instability (MSI) (30) represents a phenomenon wherein a microsatellite allele at a specific locus in a tumor arises due to the insertion or deletion of a repetitive unit, as compared to normal tissue. This marker is clinically significant due to functional defects in DNA mismatch repair mechanisms within tumor tissues.

DNA methylation constitutes a chemical alteration of DNA, characterized by the covalent attachment of a methyl group to the 5-carbon position of cytosine within a CpG dinucleotide, a process facilitated by DNA methyltransferase (31). This epigenetic modification induces alterations in chromatin structure, DNA conformation, DNA stability, and the interaction of DNA with proteins, thereby modulating gene expression. Employing the SMART (http://www.bioinfo-zs.com/smart) tool, we analyzed AEG-1 DNA methylation in tumor samples (32).

### 2.5 Correlation of AEG-1 with tumor immunity

The TISIDB database (http://cis.hku.hk/TISIDB/index.php) (33) encompasses ten principal sections dedicated to the analysis of the relationships between tumor immune cell infiltration, immunochemokines, lymphocytes, immunosuppressants, immune activators, and immune molecule subtypes. Using TISIDB, the correlation between AEG-1 and tumor immune cell infiltration and immune molecular subtypes was systematically investigated.

### 2.6 AEG-1 single-cell analysis

The CancerSEA database (http://biocc.hrbmu.edu.cn/CancerSEA/) (34) constitutes the inaugural specialized repository dedicated to comprehensively elucidating the diverse functional states of cancer cells at single-cell resolution. This resource enables the analysis of a broad spectrum of relevant cellular functions, including angiogenesis, cell cycle regulation, DNA damage response, epithelial-to-mesenchymal transition (EMT), inflammation, metastasis, gene silencing, apoptosis, differentiation, DNA repair, hypoxia, invasion, proliferation, and sensitization. The influence of AEG-1 on the biological functions of various cancer cell types was investigated using this database.

### 2.7 Cell culture and plasmid transfection

The SCC15 cell line was utilized in this study, maintained in DMEM basal medium supplemented with 10% FBS and 1% double-antibody. This approach ensured optimal growth and experimental conditions for all cell lines. Furthermore, routine monitoring for mycoplasma contamination was conducted before initiating any experimental procedures. Shanghai Gikai constructed the AEG-1 overexpressing lentivirus, the viral titer was adjusted by the lentivirus transfection manual, and puromycin selection was employed. The cell lines utilized in this study were provided by Xiu-Mei Wang, a member of our research group (35).

### 2.8 Quantitative real-time PCR

Total RNA was extracted utilizing TRIzol reagent (TAKARA, Japan). cDNA synthesis was performed using PrimeScript RT kit with gDNA Eraser (TAKARA, Japan). The expression level of the gene was quantified by real-time fluorescence quantitative PCR(RT-PCR) using SYBR Premix Ex Taq (TAKARA, Japan). The relative expression of mRNA was calculated using the 2 ΔΔCt method and normalized to the reference gene to correct for non-specific experimental variation (**Supplementary Table 1**).

### 2.9 mRNA-seq and bioinformatics analysis

Cell samples were meticulously gathered for mRNA-seq analysis after successfully constructing the AEG-1 overexpression SCC15 cell line. This component of the experiment was conducted by LC-Biotechnology Company. The data were analyzed using the company’s UNIKAWA Cloud Platform Interactive System. Per your request, the raw sequencing results have been uploaded to NutCloud (https://www.jianguoyun.com/c/sd/19a6ec9/4185bef735a28a76). Following genomic localization, the highly regarded Stringtie software (version 1.3.0) was employed to generate and annotate fragment per kilobase per million exons (FPKM) values. The threshold for statistical significance was set at a p-value corresponding to a false discovery rate (FDR) of 0.05. This resulted in the categorization of mRNAs exhibiting a 2-fold change as differentially expressed. Two groups of differentially expressed genes (DEGs) were subjected to enrichment analysis using the Gene Ontology (GO) and the Kyoto Encyclopedia of Genes and Genomes (KEGG). The immune infiltration gene set was obtained from the Immpot database; the intersection set was taken through the Venny database (https://bioinfogp.cnb.csic.es/tools/venny/index.html) (34) and the STRING database (https://cn.string-db.org/cgi/INPUT) (36) for protein network interactions. The gene clusters obtained from Cytoscape (37) were again analyzed for GO and KEGG enrichment by applying the INPUT2.0 database.

### 2.10 Protein-protein interaction docking analysis

Protein docking is a computational method to predict the interactions and mutual recognition between proteins. ZDOCK is a rigid-body protein docking algorithm that utilizes the Fast Fourier Transform Correlation technique. In contrast, RDOCK is an energy optimization process based on the CHARMm force field. RDOCK is employed to refine the binding conformations of protein-protein complexes identified by ZDOCK and to evaluate these conformations using an energy-scoring function.

### 2.11 Apoptosis staining

The cells were inoculated into 96-well plates at a predetermined concentration and cultured for 24 hours. Following incubation, the medium was removed, and the cells were washed twice with phosphate-buffered saline (PBS). Subsequently, the cells were stained according to the protocol provided by the Wanlei Annexin V-FITC/PI Apoptosis Detection Kit. The stained cells were then examined and imaged using a fluorescence-inverted microscope. The excitation wavelength for Annexin V-FITC was 488 nm, and the emission wavelength was 530 nm, resulting in green fluorescence. The excitation wavelength of propidium iodide is 488 nm, and the emission wavelength is 630 nm, resulting in red fluorescence.

### 2.12 Construction of xenograft tumors

Female BALB/c mice, aged between four and six weeks, were procured from the Beijing Viton Lever Laboratory Animal Center. To establish the xenograft tumor model, 1 × 10⁷ SCC15 cells and overexpressing AEG-1 were subcutaneously injected into the dorsal abdomen of the mice. Before the initiation of the study, a qualified veterinarian verified the health status of each mouse. The mice were housed in a controlled environment featuring a 12-hour light/dark cycle and provided ad libitum access to food and water. The subjects were randomly allocated into two groups: a control group (CTRL) and an experimental group (OV) comprising five mice. One month following the initiation of the study, all mice were humanely euthanized, and tumor tissues were harvested for further analysis. The animal experiments were conducted with the approval of the Ethics Committee of the Second Hospital of Harbin Medical University (YJSDW2023-121).

### 2.13 H&E staining

Tumor tissues were immersed in 4% PFA and fixed at room temperature. After 24 hours, the tissues were dehydrated using a tissue processor. Subsequently, the tissues were embedded in paraffin wax and sectioned into continuous thin slices 4μm thick. The sections were then baked in an oven at 60°C for 1h, followed by a dewaxing procedure to remove residual wax. Following hydrochloric acid-alcohol differentiation, the samples were rinsed with distilled water and stained with hematoxylin for 5min and eosin for 2min. The sections were then mounted using neutral gum, examined microscopically, and photographed for documentation.

### 2.14 PAS staining

The slices should be subjected to baking at 60°C for 1h. A dewaxing process and subsequent washing with distilled water should follow this. After that, staining should be conducted using the protocol specified in the PAS kit supplied by BASO. Subsequently, the slices should be sealed with neutral resin, observed under a microscope, and photographed.

### 2.15 Masson staining

The tissue sections were subjected to a baking process at 60°C for 1h, then dewaxed with water and washed with distilled water. Staining was performed using the protocol outlined in the Masson Staining Kit (Solebel). Post-staining, the sections were sealed with neutral resin, examined microscopically, and documented through photomicrography.

### 2.16 Immunohistochemical staining

Antigen retrieval was performed using an autoclave following the dewaxing of tissue sections in water. The sections were then allowed to return to room temperature and treated with 3% hydrogen peroxide for 10min. 3%BSA was applied to block non-specific binding sites. The sections were then incubated overnight with the primary antibody at 4°C (**Supplementary Table 2**). The following day, the sections were incubated with the secondary antibody at ambient temperature for one hour. Positive signals were visualized in brown using DAB as a chromogenic substrate. The nuclei were counterstained with hematoxylin for 4min. After that, the sections were mounted with a neutral dendrimer, examined under a microscope, and photographed for documentation.

### 2.17 Statistical methods

The statistical analysis and generation of figures were conducted using the R language, version 4.0.2, and GraphPad Prism 9.0. In cases where the objective is to compare continuous variables between two groups, the decision between the Student *t*-test and the Mann-Whitney test is contingent upon the specific conditions at hand. In the case of multiple groups, either one-way ANOVA or the Kruskal-Wallis test with subsequent multiple comparisons was employed, contingent on the circumstances. The prognostic significance of categorical variables was determined using the log-rank test. The threshold for statistical significance was set at a *P*-value.

## 3 Results

### 3.1 Expression of AEG-1 in tumors and its prognostic value

**Figure 1:**
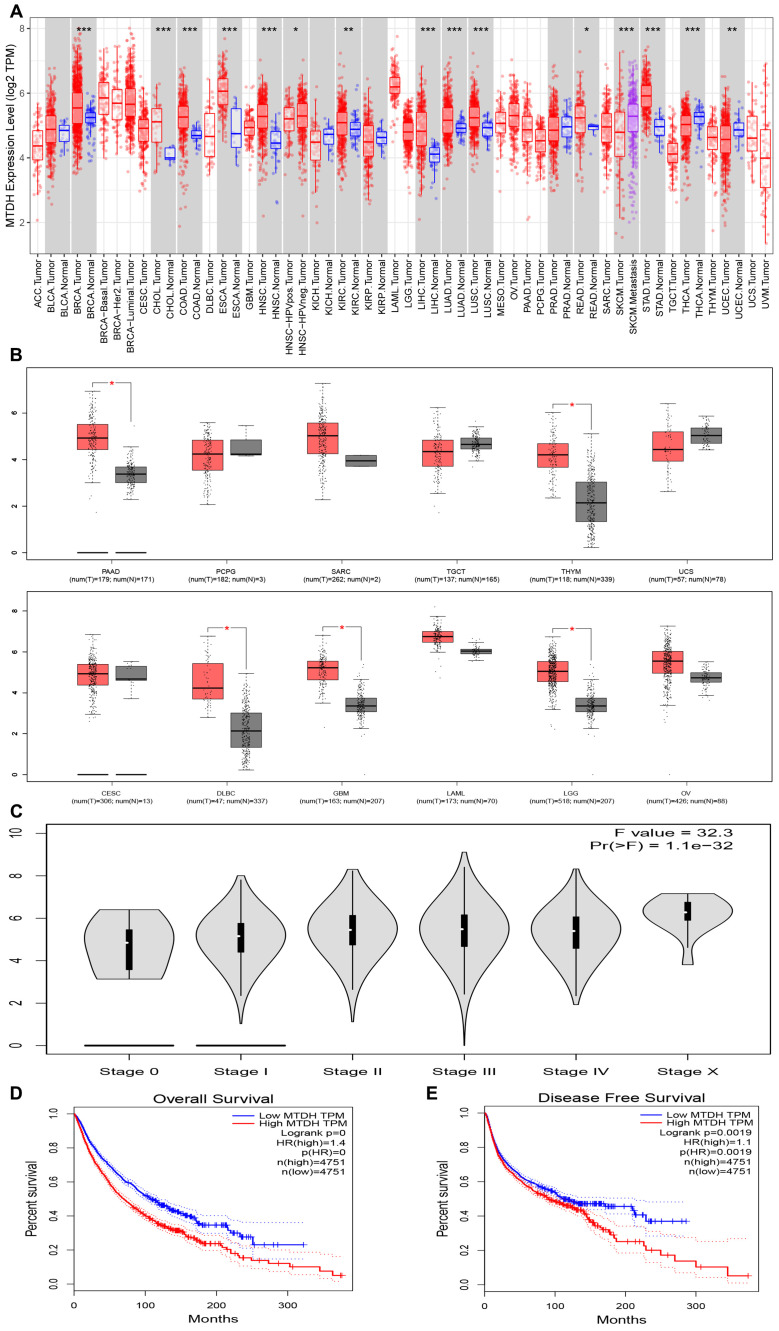
AEG-1 expression in pan-cancer from different databases. **(A)** AEG-1 expression in pan-cancer in TIMER. **(B)** AEG-1expression in PAAD, PCPG, SARC, TGCT, THYM, UCS, CESE, DLBC, GBM, LAML, LGG, and OV from the GEPIA database. **(C)** Correlation between AEG-1 and tumor grading. **(D, E)** Correlation between AEG-1 and patients’ overall survival (OS) and disease-free survival (DFS). (*, P<0.05; **, P<0.01; ***, P<0.001.).

To investigate the expression of AEG-1 in tumors, we analyzed the expression of AEG-1 mRNA in 33 cancer types using the TIMER database. The results indicated that AEG-1 was highly expressed in Breast invasive carcinoma (BRCA), Cholangio carcinoma (CHOL), Colon adenocarcinoma (COAD), Esophageal carcinoma (ESCA), HNSC, Kidney Renal Clear Cell Carcinoma (KIRC), Liver hepatocellular carcinoma (LIHC), Lung adenocarcinoma (LUAD), Lung squamous cell carcinoma (LUSC), Rectum adenocarcinoma (READ), and Stomach adenocarcinoma (STAD), and exhibited low expression in Thyroid carcinoma (THCA) and Uterine Corpus Endometrial Carcinoma (UCEC) (**Figure 1A**). Due to the unavailability of normal tissue controls for Cervical squamous cell carcinoma (CESC), Lymphoid Neoplasm Diffuse Large B-cell Lymphoma (DLBC), Glioblastoma Multiforme (GBM), Acute Myeloid Leukemia (LAML), Brain Lower Grade Glioma (LGG), Ovarian serous cystadenocarcinoma (OV), Pancreatic adenocarcinoma (PAAD), Pheochromocytoma and Paraganglioma (PCPG), Sarcoma (SARC), Testicular Germ Cell Tumors (TGCT), Thymoma (THYM), and Uterine Carcinosarcoma (UCS) in the TIMER database, we conducted an additional search in the GEPIA database. Notably, AEG-1 exhibited high expression levels in LGG, PAAD, and THYM. However, no significant correlation was observed for CESC, LAML, OV, PCPG, SARC, TGCT, and UCS (**Figures 1B, C**). This evidence suggests that the abnormal expression of AEG-1 plays a role in tumorigenesis, with predominantly deleterious effects.

The prognosis is influenced by the tumor type, the patient’s physical condition, the accessibility of suitable therapeutic interventions, and the promptness with which these interventions are administered. This study investigated the prognostic significance of AEG-1 and various tumors utilizing the GEPIA and Home for Researchers databases. We found that AEG-1 was associated with tumor grade (**Figure 1D**), negatively correlated with overall OS and DFS in tumor patients (**Figure 1E**), and was associated with OS in LGG, PAAD, Kidney Chromophobe (KICH), and KIRC, DFS in PAAD and Kidney renal papillary cell carcinoma (KIRP), and DFS in LGG, PAAD, and KIRC. KIRP, DFS in LGG, PAAD, STAD, COAD, Bladder Urothelial Carcinoma (BLCA), KIRC, KIRP, Uveal Melanoma (UVM), and Mesothelioma (MESO), and PFS in LGG, PAAD, STAD, BLCA, KICH, KIRP, UVM, and Adrenocortical carcinoma (ACC) (**Supplementary Figure 1**), and DMFS, RFS, and DSS in breast, lung, and eye cancers (**Supplementary Figure 2**). AEG-1 may become a novel tumor prognostic assessment index.

### 3.2 AEG-1 gene alterations in HNSC and other cancers

**Figure 2:**
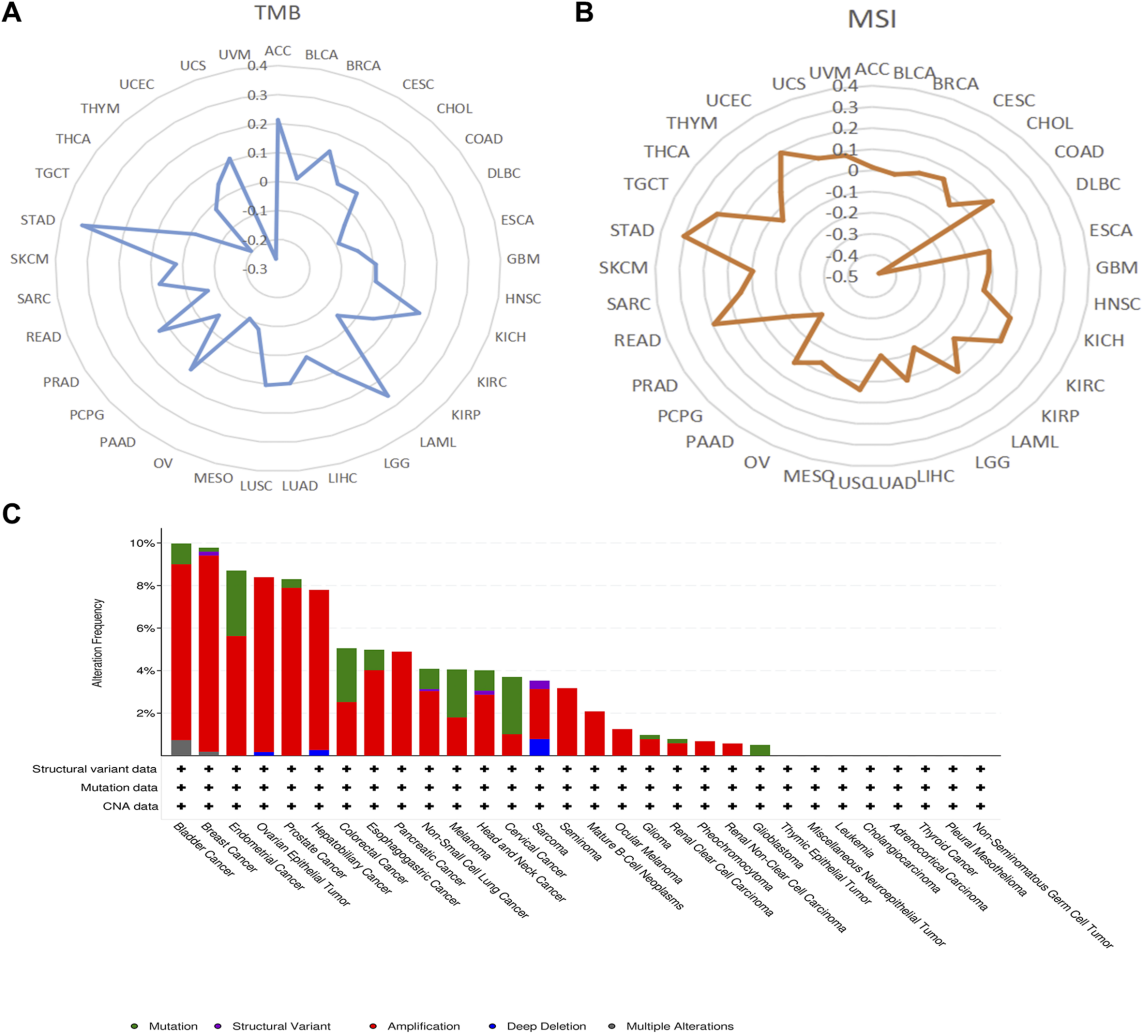
The correlation of AEG-1 expression with TMB **(A)**, MSI **(B)**, and genetic alterations of AEG-1 in pan-cancer using the cBioPortal database **(C)**.

Our investigation demonstrated that AEG-1 exhibits a significant positive correlation with TMB and a negative correlation with THCA and UVM in BRCA, LAML, LGG, LUAD, LUSC, Prostate adenocarcinoma (PRAD), and STAD (**Figure 2A**). AEG-1 was positively correlated with MSI in COAD, KIRC, READ, STAD, TGCT, and UCEC. It is negatively correlated in DLBC, LGG, LUAD, PCPG, and PRAD (**Figure 2B**).

**Figure 3:**
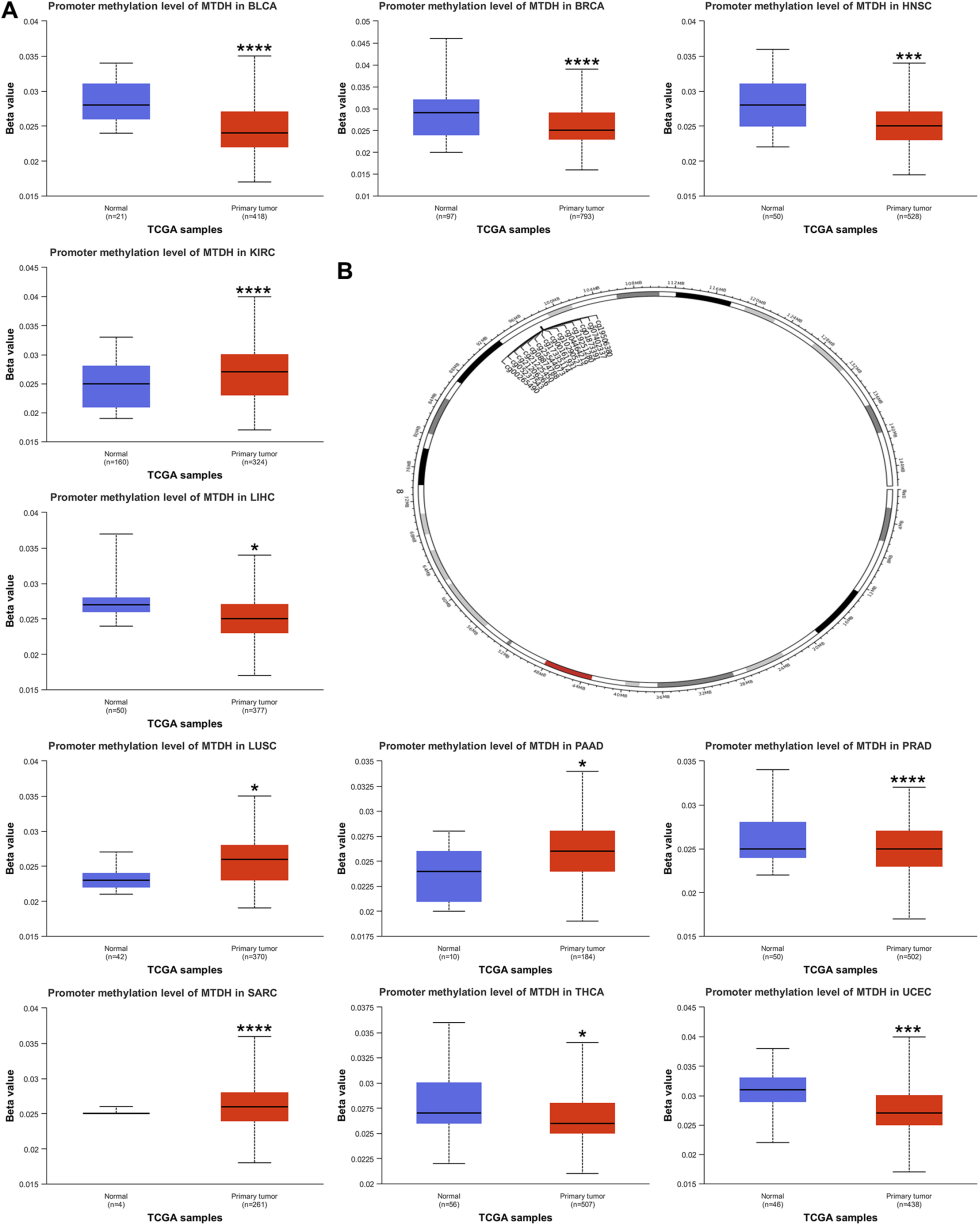
Relationship of AEG-1 with methylation. **(A)** Promoter methylation level of BLCA, BRCA, HNSC, KIRC, LIHC, LUSC, PAAD, PRAD, SARC, THCA and UCEC. **(B)** Chromosomal distribution of the methylation probes associated with AEG-1. (*, *P*<0.05; ***, *P*<0.001; ****, *P*<0.0001.).

We also discussed the genetic alterations of AEG-1 in pan-cancer using the cBioPortal database. The frequency of AEG-1 deletion was highest in BRCA (9.23%), which was mainly an amplification mutation, and the mutation was highest in UCEC (3.07), and the second and third highest frequencies of AEG-1 were found in CESC (2.69%) and COAD (2.53%) (**Figure 2C**). Meanwhile, we examined the DNA methylation levels of various tumors. The results showed that the methylation levels of KIRC, LUSC, PAAD, PRAD, and SARC were higher than those of normal tissues, and the methylation levels of BLCA, BRCA, HNSC, LIHC, THCA, and UCEC were lower than those of normal tissues (**Figure 3A**). Next, DNA methylation level and survival analysis were performed for each CpG site of AEG-1 using SMART (**Figure 3B**). AEG-1 was found to have 14 methylation probes, such as cg19506380, cg07402337, cg01873391, cg19251280, cg04464219, cg10290527, cg00161931, cg17335114, cg25544073, cg08874788, cg21225450, cg21206266, cg05237543, cg00265490.

### 3.3 Correlation of AEG-1 with tumor immune cell infiltration, immune subtypes, and molecular subtypes

Tumor-infiltrating immune cells are infiltrating immune cells isolated from tumor tissue. A successful anti-tumor immune response requires the presence, activation, and co-stimulation of all lymphoid-like components of the immune system, including various populations of T cells, B cells, DCs, NK cells, MDSCs, neutrophils, and macrophages. Our analysis showed that AEG-1 was associated with immune cell infiltration in various cancers, from T cell NK to CD^4+^Th1 and T cell CD^4+^Th2 (**Supplementary Figure 3A**). We also evaluated the association of AEG-1 with stromal scores, microenvironment scores, and immune scores in 33 cancers. We found that AEG-1 was associated with ACC, BLCA, BRCA, DLBC, LGG, LIHC, LUAD, LUSCPRAD, READ, SARC, STAD, THCA, THYM, UCEC, and UVM were positively correlated with stromal scores and negatively correlated with stromal scores in TGCT, in ACC, BRCA, CESC, ESCA, HNSC, LIHC, LUAD, LUSC, SARC, Skin Cutaneous Melanoma (SKCM), STAD, TGCT, THCA, and UCEC were positively correlated with microenvironment scores and negatively correlated with UVM; positively correlated with immune scores in CESC, ESCA, HNSC, KIRP, LUAD, LUSC, SARC, SKCM, STAD, TGCT, THCA, UCEC, and negatively correlated in LGG, PCPG, PRAD, UVM. There was no correlation with a stromal score, microenvironment score, and immunity score in CHOL, COAD, GBM, KICH, KIRC, LAML, MESO, OV, PAAD, and UCS.

Furthermore, our analysis revealed that AEG-1 is correlated with the immune subtypes of BLCA, BRCA, COAD, GBM, KIRC, LIHC, LUSC, OV, SKCM, STAD, TGCT, and UCEC (**Supplementary Figure 3B**), as well as with the molecular subtypes of BRCA, COAD, HNSC, KIRP, LGG, OV, and STAD (**Supplementary Figure 3C**). These findings unequivocally suggest that AEG-1 is intricately linked to tumor immunity.

To further investigate the potential role of AEG-1 in tumors, we examined the function of AEG-1 at the single-cell level using CancerSEA (**Supplementary Figure 4**). The results showed that AEG-1 was negatively correlated with metastasis, differentiation, proliferation, inflammation, EMT, and angiogenesis in AML, DNA damage in LUAD, apoptosis in MEL, and invasion in OV, and positively correlated with stemness in MEL.

### 3.4 Expression, clinical characteristics, correlation and immune cell infiltration of AEG-1 in HNSC

**Figure 4:**
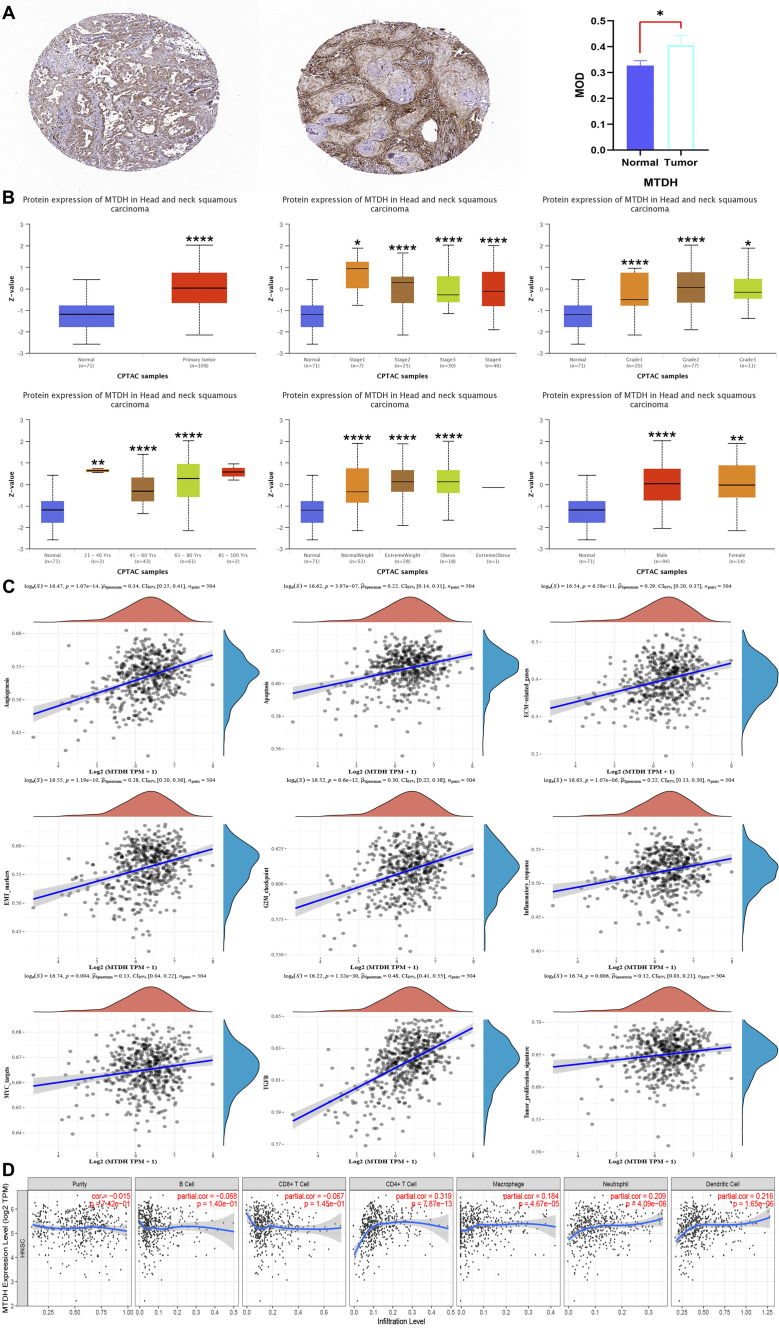
AEG-1 expression, clinical features, correlation and immune cell infiltration in HNSC. **(A)** AEG-1 expression in HNSC from the HPA database, normal tissue on the left, HNSC tissue in the middle and quantitative analysis on the right. **(B)** AEG-1 was correlated with HNSC patients’ grading, staging, age weight and gender correlation. **(C)** Correlation of AEG-1 with angiogenesis, apoptosis, EMC-related genes, EMT markers, G2M checkpoints, immune response, MYC markers, and tumor proliferation in HNSC. **(D)** Correlation of AEG-1 with immune infiltration of HNSC. n=5. (*, *P*<0.05; **, *P*<0.01; ****, *P*<0.0001.).

It is established that AEG-1 is closely associated with HNSC, but its correlation with immune cell infiltration in HNSC has not been investigated. Our study confirmed that AEG-1 was highly expressed in OSCC by HPA (**Figure 4A**); UALCAN discussed the correlation between AEG-1 and clinical characteristics of HNSC and found that AEG-1 was with higher correlation with females than males, and closer correlation with age, body weight, and grading with increasing (**Figure 4B**). Additionally, AEG-1 demonstrated a correlation with angiogenesis, apoptosis, extracellular matrix-related genes, epithelial-mesenchymal transition markers, G2/M checkpoints, immune response, MYC markers, and tumor proliferation in HNSC (**Figure 4C**).

**Figure 5:**
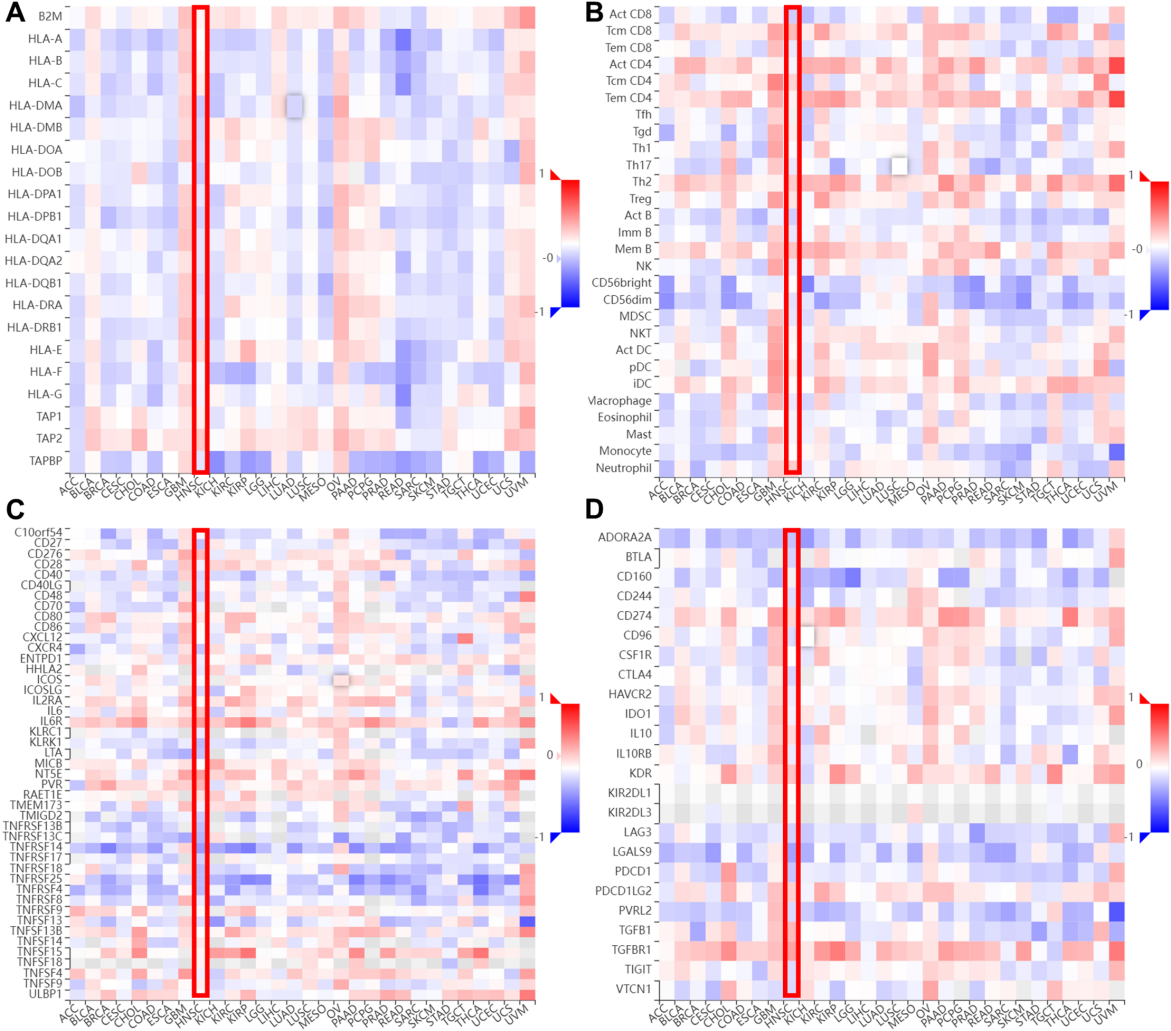
The relevance between AEG-1 and chemokines in different cancer types. **(A)**The relevance between AEG-1 methylation status and MHC molecules. **(B)** The relevance between AEG-1 methylation status and Lymphocyte. **(C)** The relevance between AEG-1 methylation status and Immunostimulator. **(D)** The relevance between AEG-1 methylation status and Immunoinhibitor.

We also explored the correlation between AEG-1 and immune cell infiltration in HNSC through the TIMER database, and AEG-1 was correlated with CD^4+^ T cells (*P*=7.87e-13), macrophage (*P*=4.67e-05), neutrophil (*P*=4.09e-06), and DCs (*P*=1.65e-06) were significantly positively correlated, with no correlation with B cell and CD^8+^ T cell (**Figure 4D**). In addition, we analysed other immune-related cells (**Supplementary Table 3**). To further explore the correlation of AEG-1 with immune molecules, immunostimulatory molecules, immunosuppressive molecules, immunochemokines, and lymphocytes, we also searched by TSIDB (**Figures 5A–D**). We found that it was correlated with tumor immunotherapy targets BTLA, CTLA4, HAVCR2, LAG3, PDCD1, and TIGIT in HNSC; ICOS were all significantly correlated.

### 3.5 Identification and analysis of differentially expressed genes associated with AEG-1 in OSCC

**Figure 6:**
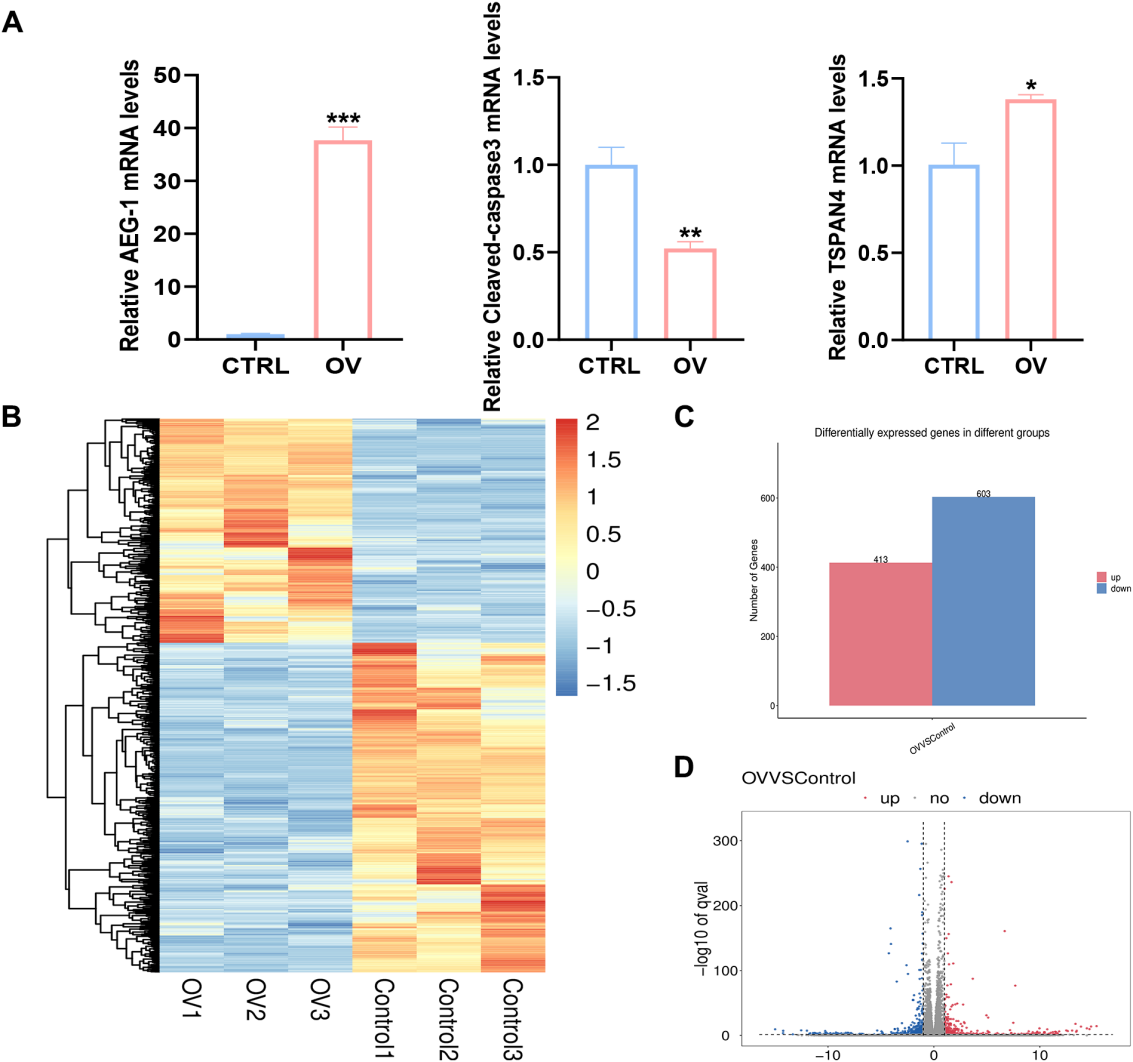
AEG-1 transcriptomic analysis after overexpression. **(A)** RT-PCR to detect the correlation of AEG-1 with Caspase3, TSPAN4 **(B)** Heatmap of Hierarchical clustering analysis of changed mRNAs. **(C, D)** mRNAs differentially expressed between CTRL and OV group. (*, *P*<0.05; **, *P*<0.01; ***, *P*<0.001.).

**Figure 7:**
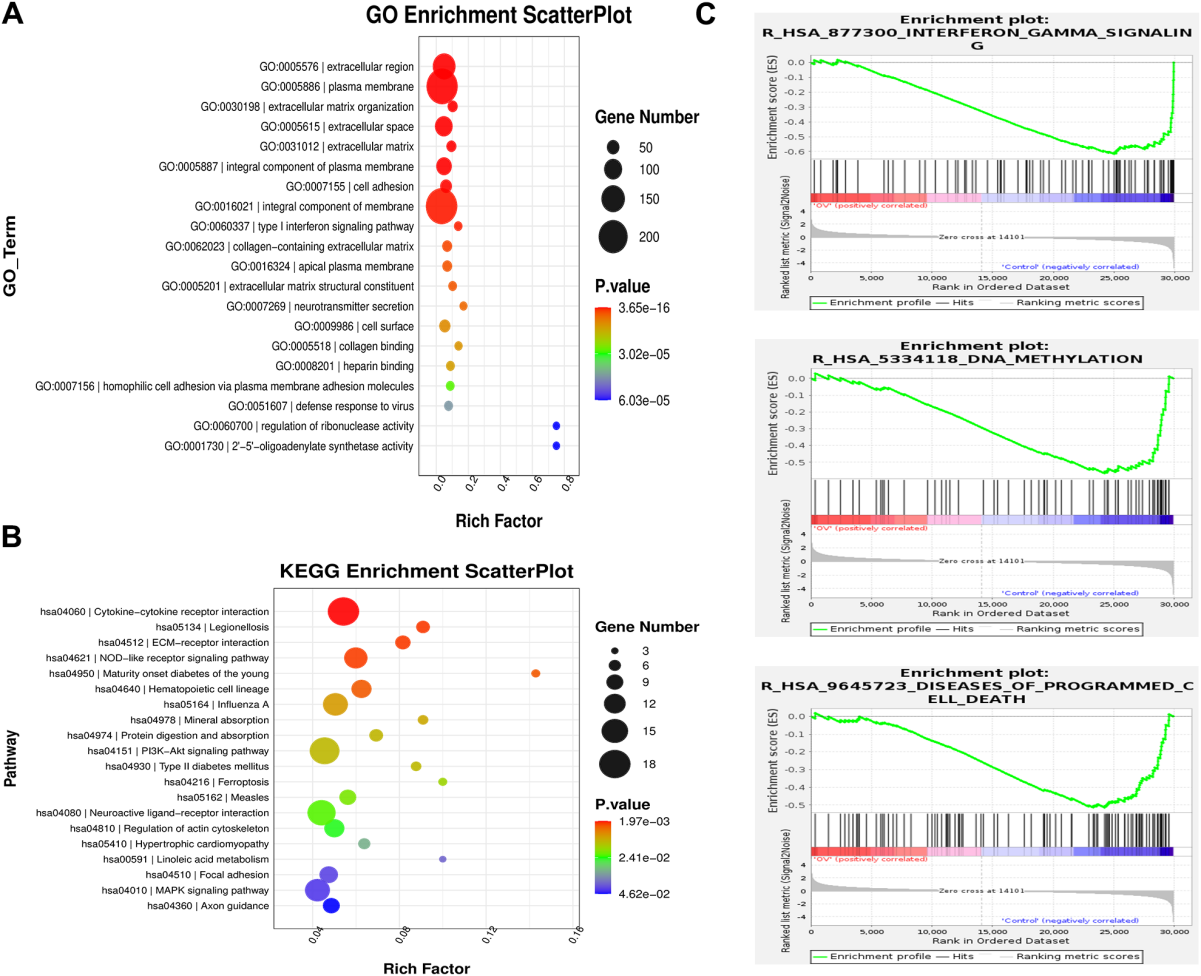
AEG-1-related gene enrichment analysis. **(A)** GO analysis of the AEG-1 binding proteins. **(B)** KEGG analysis of the AEG-1 binding proteins. **(C)** GSEA analysis of the AEG-1 binding proteins.

To further elucidate the correlation between AEG-1 and immune cell infiltration in OSCC, we engineered OSCC cell lines with overexpressed AEG-1. Our findings indicate that AEG-1 overexpression enhances the expression of SCC15 migration markers while inhibiting apoptosis (**Figure 6A**). Additionally, mRNA-seq analysis revealed 1016 differentially expressed genes, comprising 413 up-regulated and 603 down-regulated genes (**Figures 6B–D**). We also performed GO enrichment analysis and found that AEG-1 was mainly localized in the extracellular matrix and cell membrane and was involved in biological processes such as cell adhesion, type I interferon signaling pathway, neurotransmitter secretion, viral defense, IFN-γ-mediated signaling pathway, angiogenesis, and immune response, angiogenesis, and immune response, and possessed 2’-5’-oligomeric adenosine monophosphate synthetase activity, peptidase inhibitor activity, transmembrane protein transporter activity, small molecule binding, CD4 receptor binding, iron ion binding, and calcium ion binding (**Figure 7A**). At the same time, KEGG was mainly enriched in cytokine-cytokine receptor interaction, Legionnaires’ disease, ECM receptor interaction, NOD-like receptor signaling pathway, PI3K-Akt signaling pathway, ferroptosis and MAPK signaling pathway, and other signaling pathways (**Figure 7B**). GSEA enrichment analysis revealed that AEG-1 was highly associated with IFN-γ, DNA methylation, and programmed cell death in OSCC (**Figure 7C**).

### 3.6 AEG-1-related expression of differential genes in OSCC is associated with immune cell infiltration

**Figure 8:**
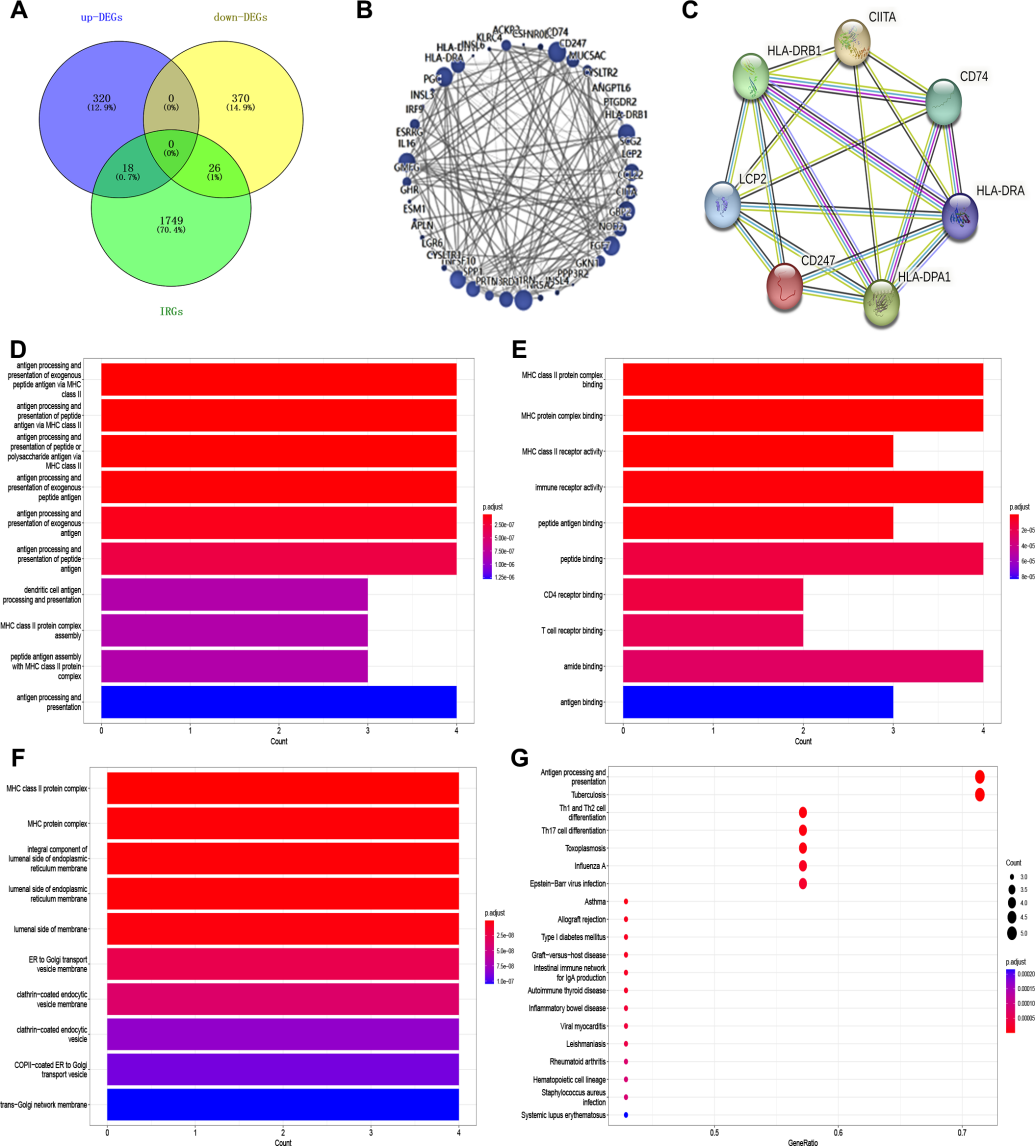
AEG-1 is associated with immune-related factors in OSCC. **(A)** Venn’s plot to obtain the intersecting genes of AEG-1 overexpressed differential genes with the set of immune-related genes. **(B)** Intersected genes were subjected to PPI. **(C)** Gene clusters were obtained by Cytoscape. **(D)** GO analysis (biological process) of the AEG-1 binding proteins. **(E)** GO analysis (cellular component) of the AEG-1 binding proteins. **(F)** GO analysis (molecular function) of the AEG-1 binding proteins. **(G)** KEGG analysis of the AEG-1 binding proteins.

We acquired the immune cell infiltration gene set, comprising 1,793 genes, from the Immpot database. Subsequently, we enriched this gene set with differentially expressed genes identified through transcriptomic analysis, resulting in 44 differential genes, of which 18 were up-regulated, and 26 were down-regulated (**Figure 8A**). Concurrently, we conducted PPI analysis using STRING on these 44 differential genes (**Figure 8B**). Further Cytoscape enrichment analysis identified 7 vital differential genes: LCP2, CD247, HLA-DPA1, HLA-DRA, HLA-DRB1, CIITA and CD74 (**Figure 8C**). Subsequently, the seven differential genes were subjected to GO and KEGG enrichment analyses, revealing significant associations with Th1/Th2 cells, Th17 cells, and other immune cell types (**Figures 8D–G**). To elucidate the interaction between AEG-1 and the seven differential genes, we conducted a protein-protein docking analysis using ZDOCK, which allowed us to determine the binding energies of AEG-1 with each of the seven differential genes (**Supplementary Figure 5**). These findings suggest that AEG-1 may serve as an immune prognostic marker for OSCC.

### 3.7 AEG-1 is associated with OSCC immunity and affects Th1/Th2 immune balance

**Figure 9:**
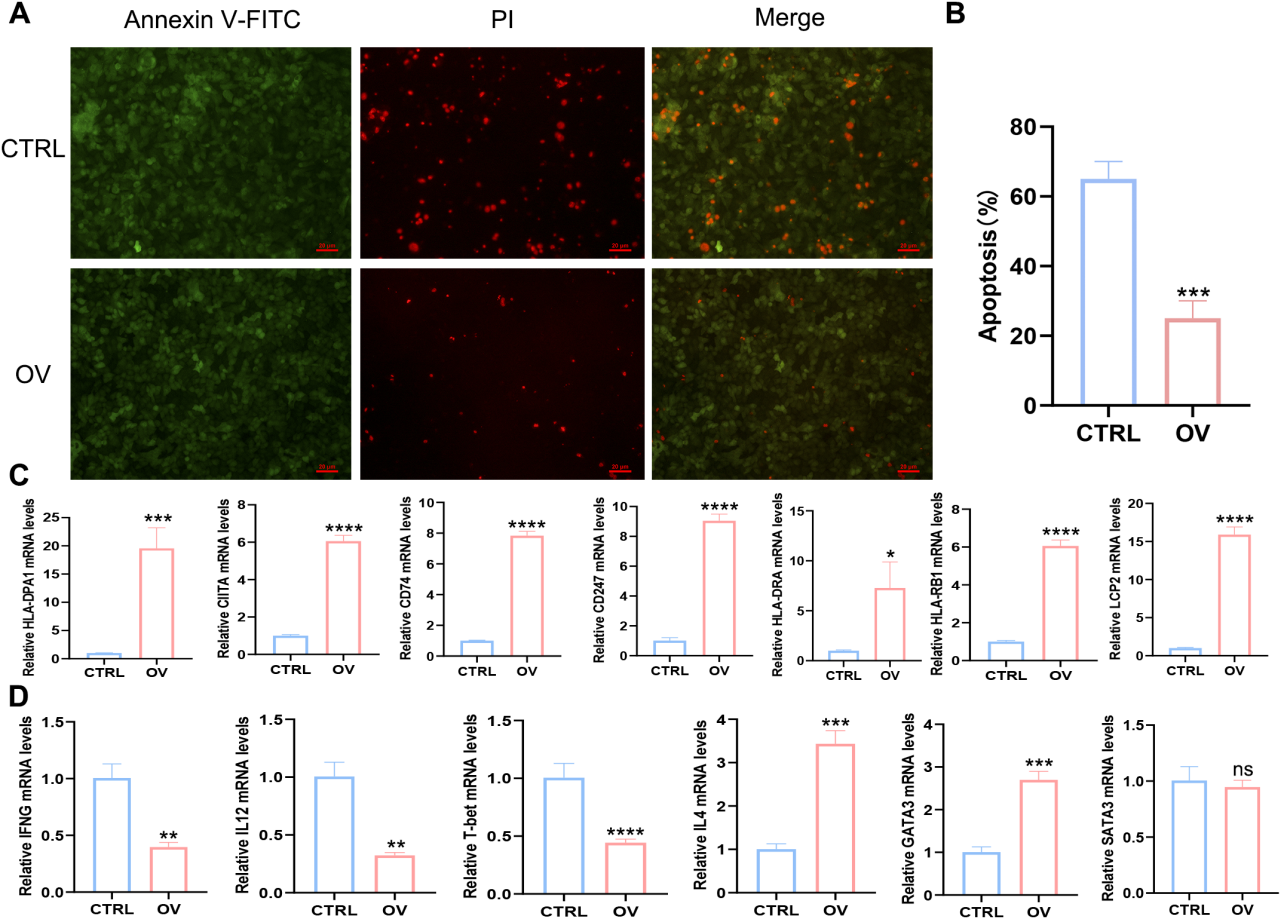
AEG-1 is associated with immunity and regulates Th1/Th2 immune homeostasis. **(A, B)** Apoptosis staining to explore the effect of AEG-1 on apoptosis. **(C)** RT-PCR to detect the correlation of AEG-1 with LCP2, CD247, HLA-DPA1, HLA-DRA, HLA-DRB1, CIITA, and CD74. **(D)** RT-PCR was performed to detect the correlation between AEG-1 and Th1-associated factors IFNG, IL12 and T-bet, and Th2-associated factors IL4, GATA3 and STAT3. (ns, *P*>0.05; *, *P*<0.05; **, *P*<0.01; ***, *P*<0.001; ****, *P*<0.0001.).

Our study demonstrated that AEG-1 induces apoptosis in SCC15 cells, as evidenced by apoptosis staining (**Figures 9A, B**). RT-PCR analysis further revealed that SCC15 cells overexpressing AEG-1 exhibited a strong association with immune-related factors, including HLA-DPA1, CIITA, CD74, CD247, HLA-DRA, LCP2, and HLA-DRB1 (**Figure 9C**). Additionally, AEG-1 overexpression promoted the expression of Th2-related factors IL4 and GATA3 while inhibiting the expression of Th1-related factors IFN-γ, IL12, and T-bet (**Figure 9D**).

**Figure 10:**
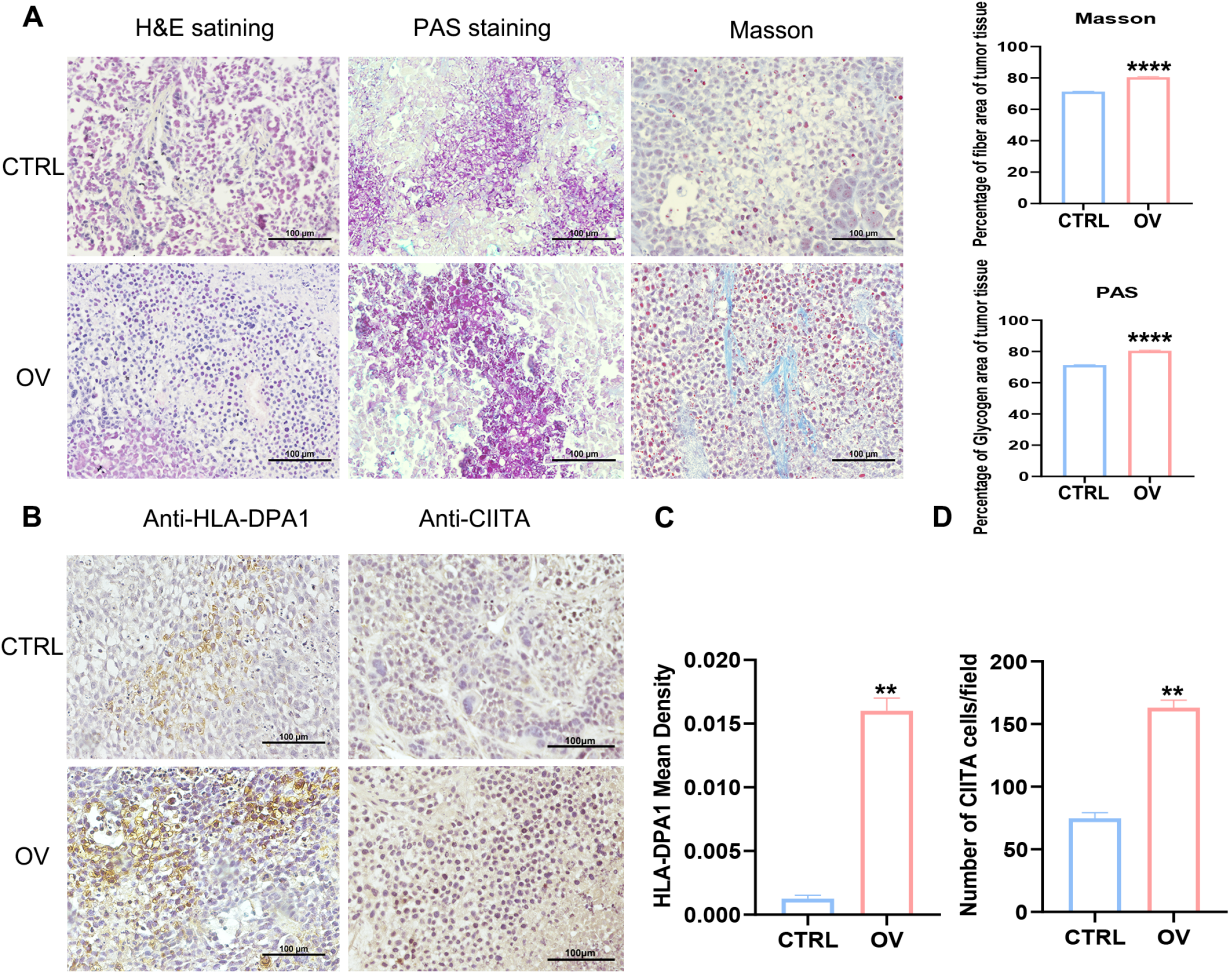
AEG-1 affects tumor immunity. **(A)** The effect of AEG-1 on tumors was explored by H&E staining, PAS staining and Masson staining. **(B–D)** Immunohistochemistry probed the correlation of AEG-1 with immune-related factors HLA-DPA1 and CIITA in tumor tissues. n=3. (**, *P*<0.01; ****, *P*<0.0001.).

The *in vivo* establishment of xenograft tumors and PAS and Masson’s staining demonstrated that AEG-1 promotes tumor growth(**Supplementary Figure 6**), significantly enhanced glycogen accumulation and fibrosis within tumor tissues compared to the control group (**Figure 10A**). Furthermore, IHC analysis revealed an up-regulation of immune-related factors HLA-DPA1 and CIITA in tumor tissues overexpressing AEG-1 (**Figures 10B–D**).

## 4 Discussion

AEG-1, an HIV- and TNF-α-inducible gene (18) in PHFAs, is predominantly localized in the endoplasmic reticulum and is implicated in the initiation, progression, and metastasis of breast (38), gastric (39), liver (8), and OSCC. This study reveals the gene expression, prognosis, alteration, immune infiltration, and DNA methylation of AEG-1 in pan-cancer, elucidating its development and prospective role. This was accomplished by utilizing the TIMER, GEPIA, and TISIDB databases derived from the TCGA, GTEx, and UALCAN repositories.

The findings of our study demonstrate that AEG-1 is markedly expressed in 16 types of cancers, whereas its expression is low in THCA and UCEC. This differential expression implies that AEG-1 may have varying functional roles across tumor types. Furthermore, AEG-1 expression is associated with OS, DFS, DSS and PFS in PAAD, suggesting its potential as a prognostic biomarker for PAAD. Additionally, prior research by our group has established that AEG-1 is significantly expressed in OSCC.

Furthermore, AEG-1 has been demonstrated to enhance the proliferation, migration, invasion, and EMT of OSCC. However, the association between AEG-1 and immune infiltration in OSCC remains inadequately understood. Consequently, this study aims to elucidate the relationship between AEG-1 and immune infiltration in both OSCC and pan-cancer contexts.

The current biomarkers associated with the efficacy of immunotherapy encompass but are not limited to, PD-L1 expression, MSI status, TMB, Epstein-Barr virus (EBV) infection status, and tumor-infiltrating lymphocytes (TILs). Our study identified a significant correlation between AEG-1 and TMB and MSI across various tumor types in a pan-cancer analysis. Notably, AEG-1 exhibited a positive association with both TMB and MSI in STAD, indicating that AEG-1 may serve as a potential immunotherapeutic biomarker for STAD. Furthermore, analyses of tumor immune cell infiltration, immunosubtyping, and molecular subtyping revealed that AEG-1 was negatively correlated with T cell NK in 27 out of 33 cancers, positively correlated with Th2 in 27 cancers, and negatively correlated with Th1 in 29 cancers. It can thus be postulated that AEG-1 may impede the secretion of Th2-associated factors by promoting the secretion of Th2-related factors, which in turn inhibits Th1 differentiation and attenuates T cell NK’s ability to regulate the recruitment and function of other immune cells through the secretion of cytokines, thus exerting a pro-tumorigenic effect.

Furthermore, we examined the association between AEG-1 and immune cell infiltration in OSCC. Our findings indicated that AEG-1 is correlated with several clinical parameters in HNSC patients, including OS, gender, age, body weight, tumor grade, and stage. Additionally, AEG-1 was found to be associated with various biological processes, such as angiogenesis, apoptosis, ECM-related genes, EMT markers, G2/M checkpoints, immune response, MYC markers, tumor proliferation, and TGF-β signaling. Furthermore, an analysis utilizing the TSIDB database uncovered a significant correlation between AEG-1 and several key factors, including the tumor-promoting cell Th2, immune activator IL6, CXCR4, immunosuppressants CTLA4, IL1, IDO1, LAG3, PDCD1, TGFB1, and the DHC molecule HLA-DPA1. These findings support the hypothesis that AEG-1 is integral to the immunotherapy of HNSC.

Research indicates that excessive glycogen accumulation, resulting from liquid-liquid phase separation, leads to the inactivation of the Hippo pathway (40). This inactivation inhibits cellular carcinogenesis and concurrently enhances the activity of the downstream proto-oncoprotein YAP, thereby promoting tumor development. Additionally, fibrosis within tumors represents the body’s response to malignancy and further contributes to tumor growth and metastasis. We developed a nude mouse tumor model through the establishment of an OSCC cell line with overexpression of AEG-1. Our findings indicate that AEG-1 facilitates glycogen accumulation and fibrosis within tumors, thereby providing compelling evidence that AEG-1 plays a significant role in promoting tumorigenesis and tumor progression.

DCs constitute a distinct class of immune cells distributed across various organs. They are classified as antigen-presenting cells owing to their capacity to present antigens—molecular structures characteristic of organ cells—to T cells, thereby initiating an immune response. Within the tumor microenvironment, dendritic cells perform the functions of antigen uptake, processing, and presentation, which subsequently activates the T-cell-mediated immune response targeting tumor cells. Due to their involvement in tumorigenesis and progression, DCs have been extensively utilized in tumor immunotherapy (41). Furthermore, dendritic cells facilitate the proliferation and maturation of B lymphocytes and activate Th cells and NK cells, thereby enhancing immune function through multiple pathways (42). Our study identified a significant correlation between AEG-1 and immune-related genes, including HLA-DPA1, CIITA, HLA-DRB1, CD74, HLA-DRA, LCP2, and CD247. This association was elucidated through an integrative approach combining mRNA-seq and biosignature analysis. HLA-DPA1, CIITA, HLA-DRB1, and HLA-DRA have been identified as critical markers for dendritic cells (DCs). CD74 and LCP2 serve as markers for lymphocytes, whereas CD247 is indicative of natural killer (NK) cells. Consequently, it can be hypothesized that AEG-1 influences immune function through multiple pathways, including the modulation of B lymphocyte proliferation and maturation, as well as the regulation of Th cell and NK cell activation via DC surface markers. Therefore, AEG-1 emerges as a potential novel target for immunotherapy in OSCC.

In recent years, a growing body of evidence has demonstrated that T-cell immunity, activated by DC vaccines (43), plays a pivotal role in the anti-tumor response and is being investigated for its potential across a spectrum of clinical applications, including melanoma, renal cancer, prostate cancer, bladder cancer, gastrointestinal tumors, gynecological tumors, and endocrine tumors, among others. These studies have indicated that DC vaccines can reduce tumor recurrence rates and enhance patient survival outcomes. The data from a real-world study of Provenge (sipuleucel-T), the first dendritic cell vaccine approved in the United States, has been published. This study examined the survival outcomes of patients with metastatic castration-resistant prostate cancer (mCRPC) who received the cancer therapeutic vaccine Provenge in conjunction with oral medication in a real-world clinical setting. The findings indicated a 45% reduction in mortality risk among prostate cancer patients, alongside an extension in OS by 14.5 months. In summary, AEG-1 is closely linked to DC and disrupts immune function through multiple mechanisms. Consequently, a DC-based vaccine may represent a promising therapeutic strategy for OSCC.

## Data Availability

The original contributions presented in the study are included in the article/**Supplementary Materials**, further inquiries can be directed to the corresponding author.
