# Supplementary material for: mRNA-seq-based analysis predicts: AEG-1 is a therapeutic target and immunotherapy biomarker for pan-cancer, including OSCC
Source: Front Immunol. 2024 Oct 17;15:1484226. doi: 10.3389/fimmu.2024.1484226 (PMC11524818; doi:10.3389/fimmu.2024.1484226)
Supplement: Supplementary file 1 [file DataSheet1.pdf]

Supplementary Material

1 Supplementary Figures and Tables

1.1 Supplementary Figures

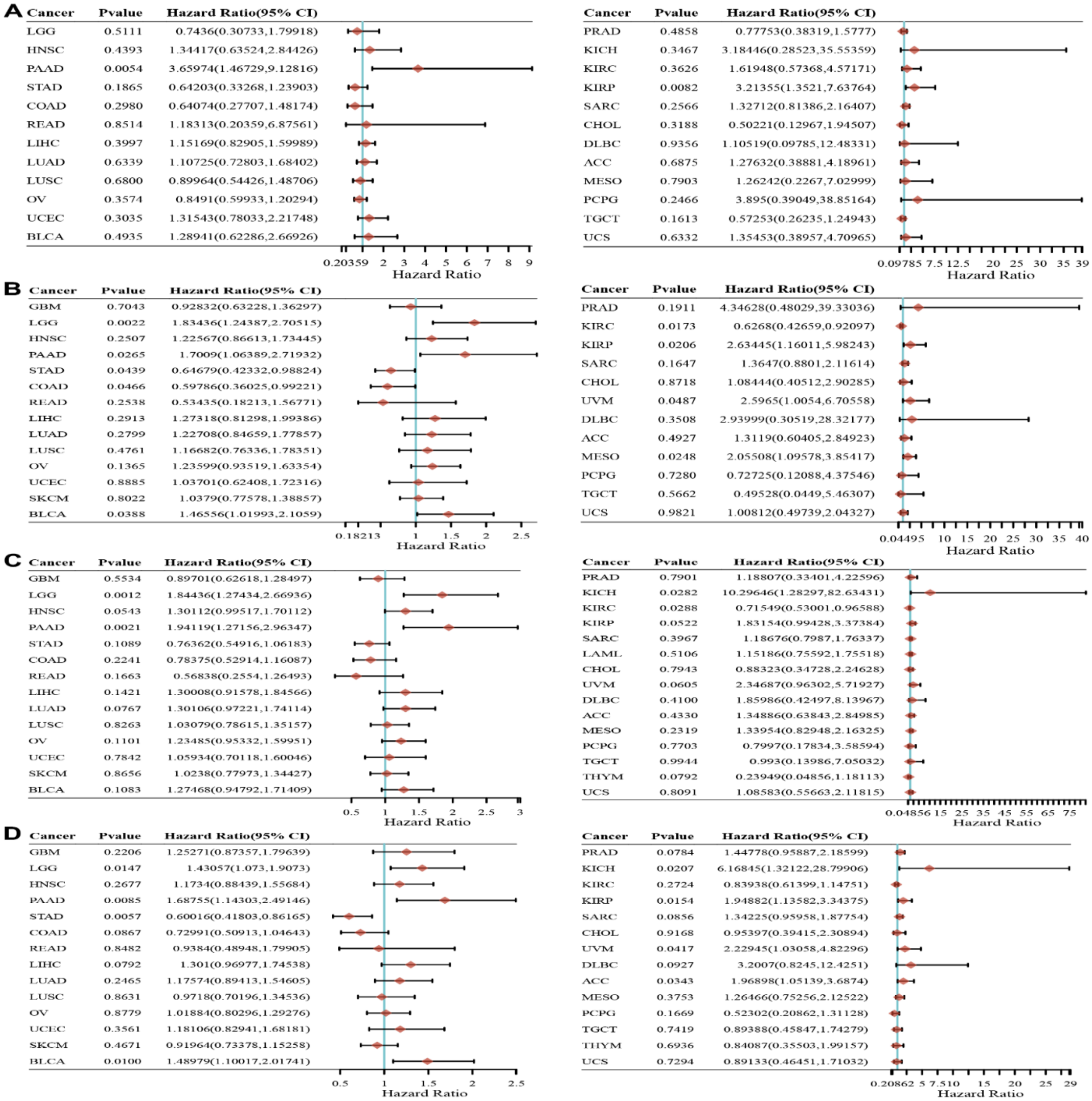

Supplementary Figure 1. The prognostic significance of AEG-1 in human tumors. (A) Univariate Cox regression of AEG-1 expression level for disease free interval. (B) Univariate Cox regression of AEG-1 expression level for disease specific survival. (C) Univariate Cox regression of AEG-1 expression level for overall survival. (D) Univariate Cox regression of AEG-1 expression level for progression free interval.

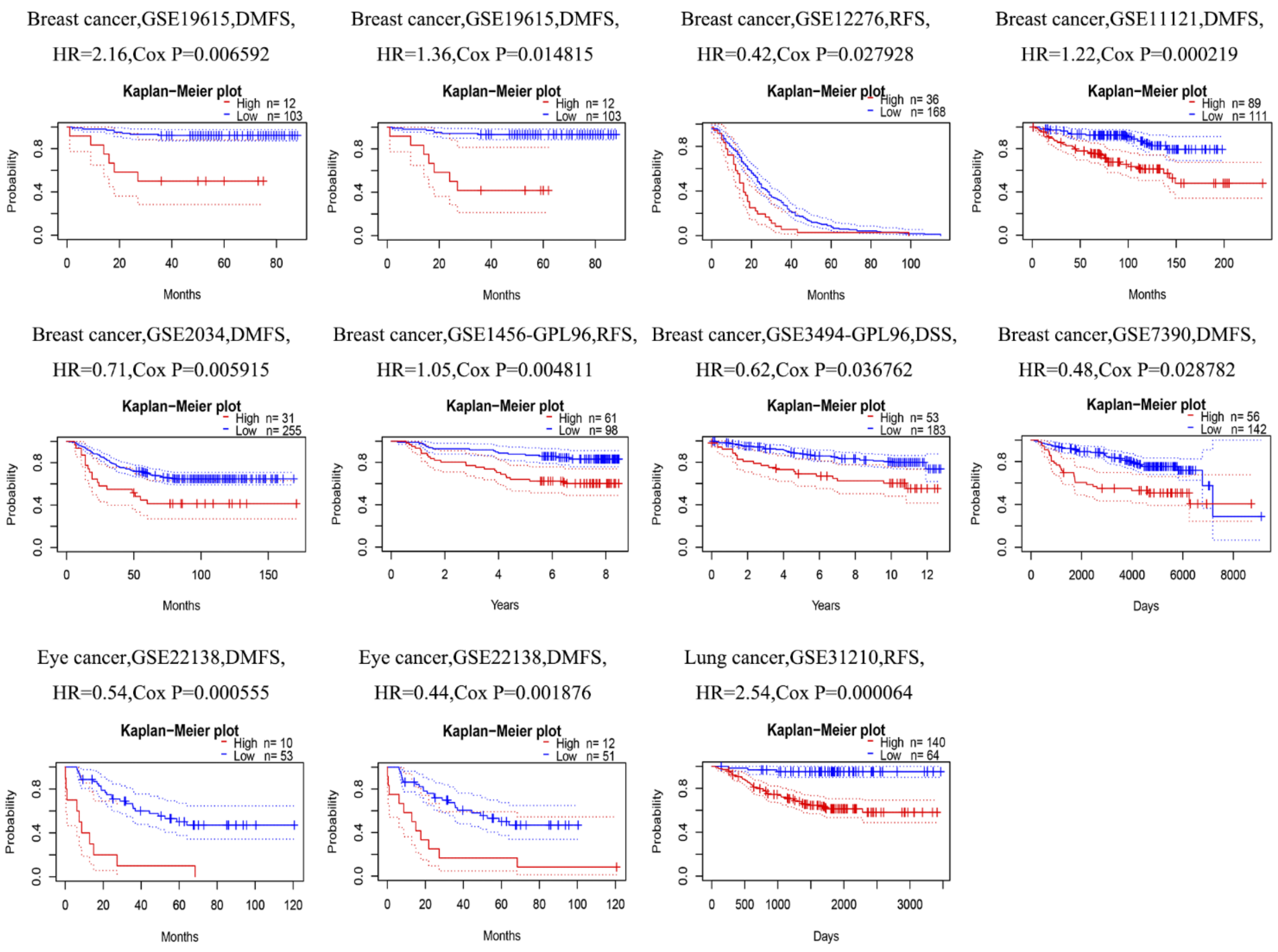

Supplementary Figure 2. Kaplan–Meier survival curves comparing high and low expression of AEG-1 in different cancer types in PrognoScan.

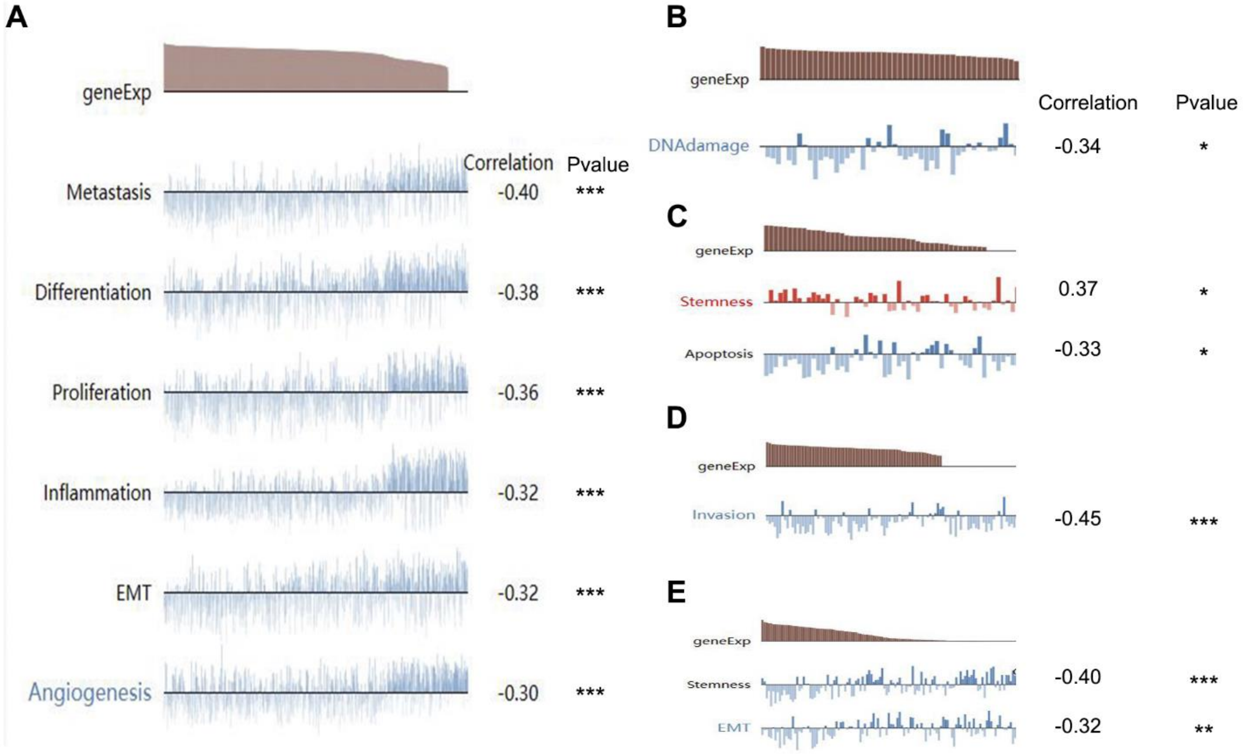

Supplementary Figure 2. The function of AEG-1 in single-cell functional analysis from the CancerSEA database. (A) Correlation analysis between functional status and AEG-1 in AML. (B) Correlation analysis between functional status and AEG-1 in LUAD. (C) Correlation analysis between functional status and AEG-1 in MEL. (D) Correlation analysis between functional status and AEG-1 in OV. (E) Correlation analysis between functional status and AEG-1 in PC. (*, P<0.05; **, P<0.01; ***, P<0.001.)

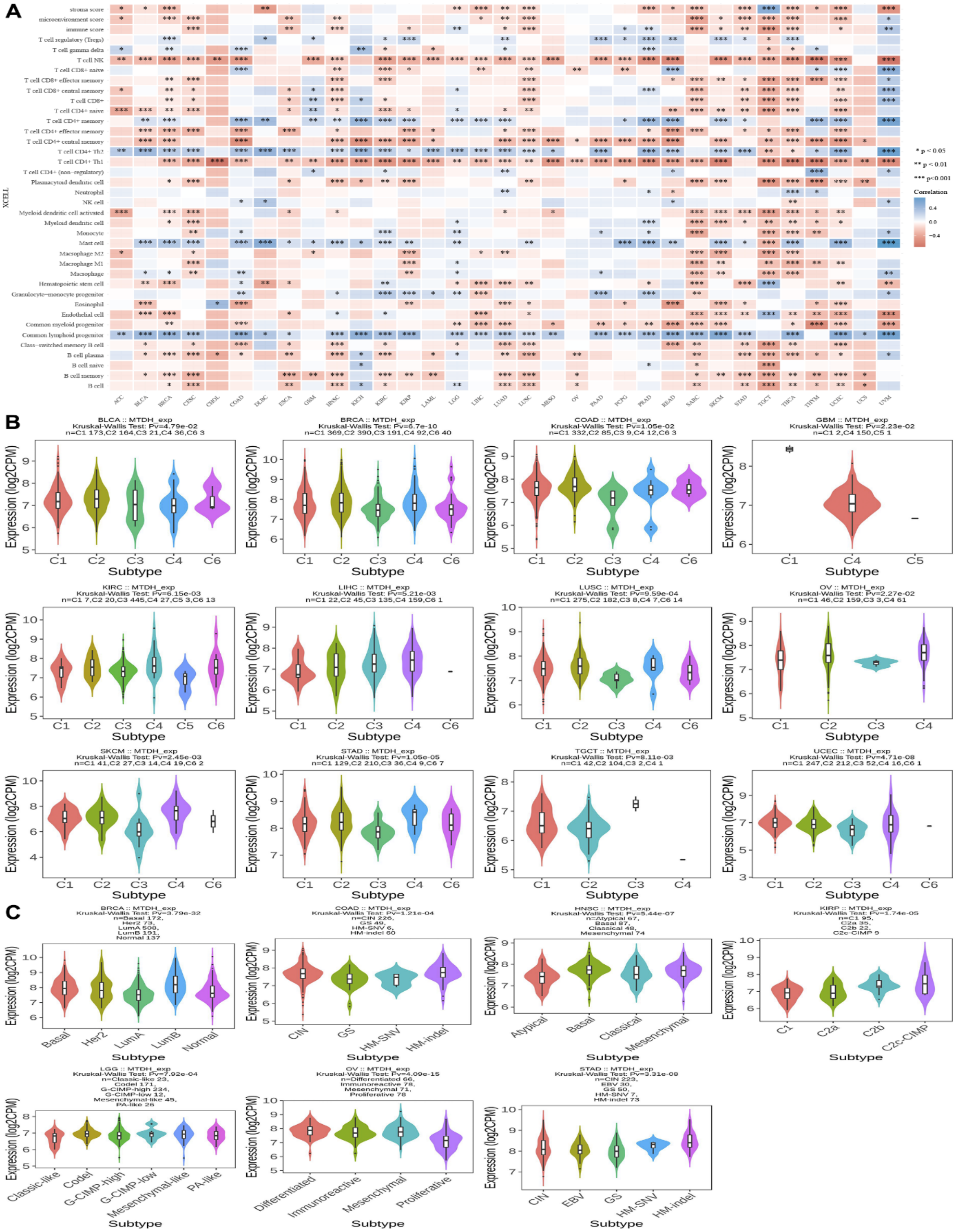

Supplementary Figure 3. Correlation between AEG-1 and immune cells, immune subtypes in pan–cancer. (A) Heat map of AEG-1 expression correlation with 33 tumor infiltrating cells.(B) The relevance between AEG-1 expression and pan-cancer immune subtypes.(C) The relevance between AEG-1 expression and pan-cancer Molecular subtype. (*, P<0.05; **, P<0.01; ***, P<0.001.)

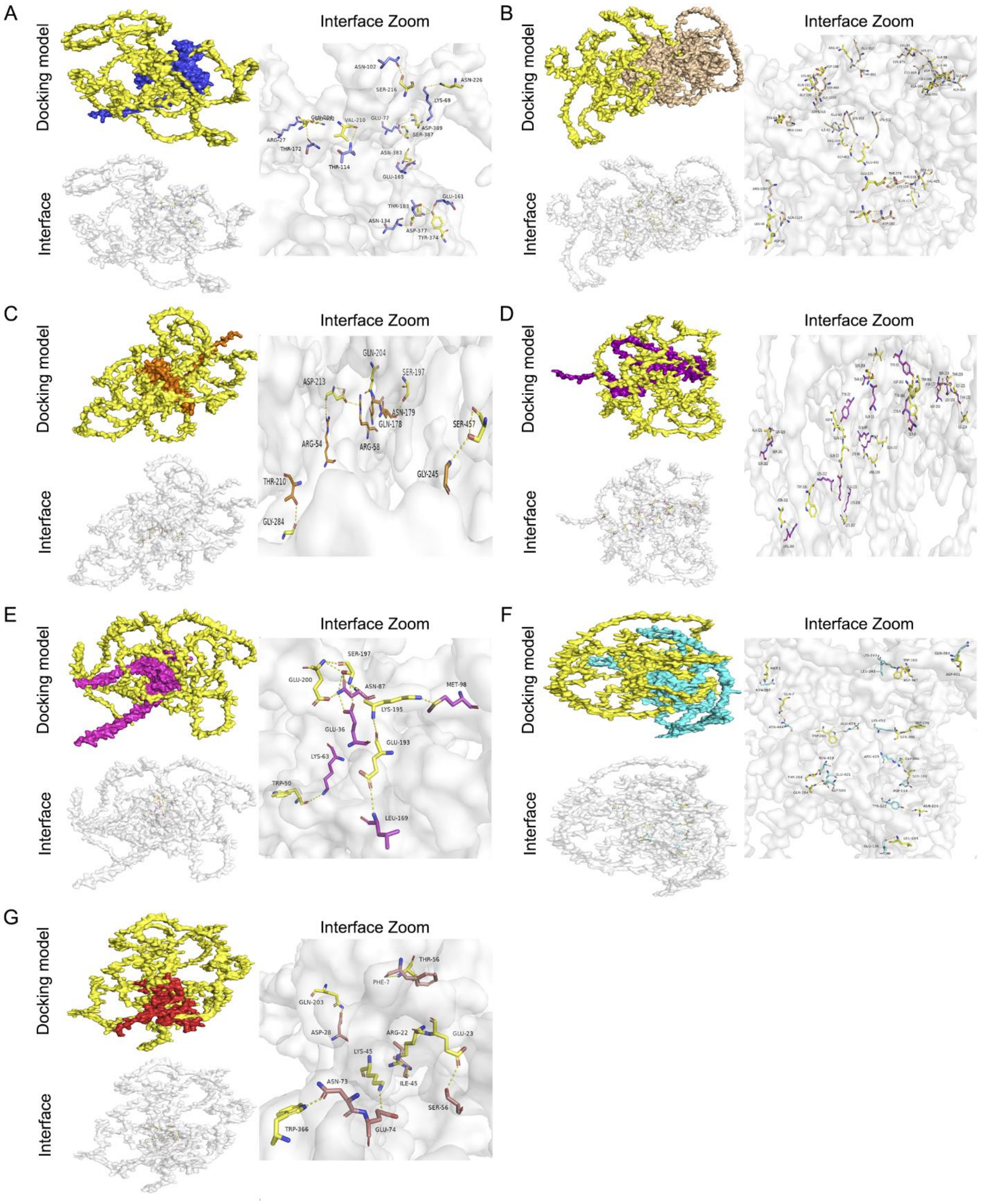

Supplementary Figure 4. Protein-protein docking prediction of AEG-1 with the immune-associated factors LCP2, CD247, HLA-DPA1, HLA-DRA, HLA-DRB1, CIITA and CD74.

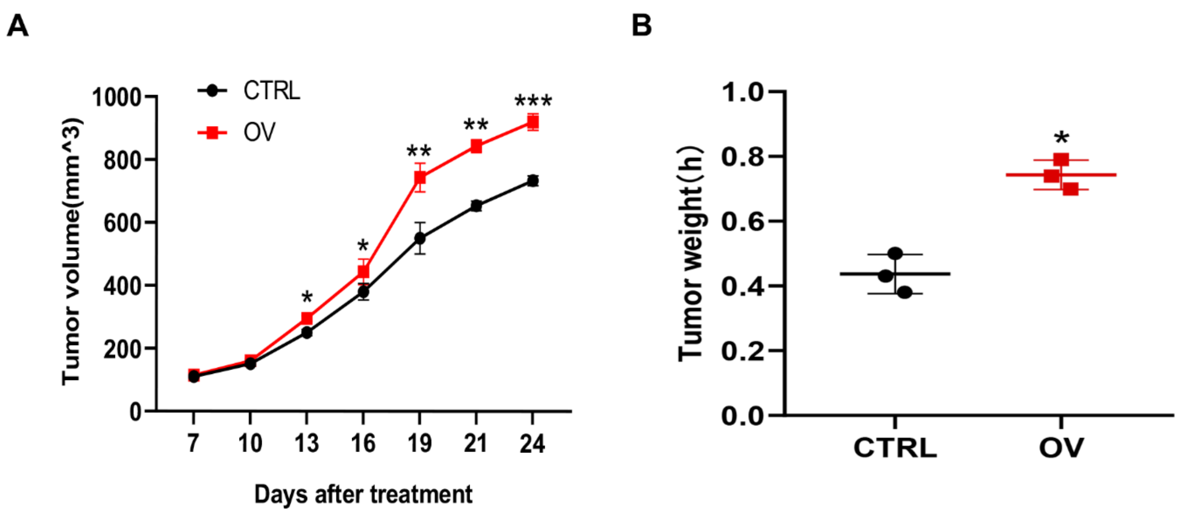

Supplementary Figure 5.(A) Effect of AEG-1 on tumor volume in Balb/c mice bearing SCC15 cells. (B) Effect of AEG-1 on tumor weight in balb/c mice bearing SCC15 cells and quantitative analysis. Data presented as mean ± SD. Significance was calculated with Student's t test. (*, $P<0.05$; **, $P<0.01$; ***, $P<0.001$.)

1.2 Supplementary Tables

Supplemental Table 1. Primers for PCR used

Gene	Forward primer(5'-3')	Reverse primer(5'-3')
AEG-1	AAATGGGCGGACTGTTGAAGT	CTGTTTTGCACTGCTTTAGCAT
Caspase3	CATGGAAGCGAATCAATGGACT	CTGTACCAGACCGAGATGTCA
TSPAN4	GCTGTGGCGTCTCCAACTAC	CTTGGCAGTACATGGTCATGG
HLA-DAP1	ATGCGCCCTGAAGACAGAATG	ACACATGGTCCGCCTTGATG
CIITA	CCTGGAGCTTCTTAACAGCGA	TGTGTCGGGTTCTGAGTAGAG
CD247	GGCACAGTTGCCGATTACAGA	CTGCTGAACTTCACTCTCAGG
CD74	GATGACCAGCGCGACCTTATC	GTGACTGTCAGTTTGTCCAGC
LCP2	GAGGAGCATCTTCACACGCAA	CGGCTCATAATCCGCGTCAT
HLA-DRB1	CGGGGTTGGTGAGAGCTTC	AACCACCTGACTTCAATGCTG
HLA-DRA	AGTCCCTGTGCTAGGATTTTTCA	ACATAAACTCGCCTGATTGGTC
IFNG	TCGGTAACTGACTTGAATGTCCA	TCGCTTCCCTGTTTTAGCTGC
IL12	GCGGAGCTGCTACACTCTC	CCATGACCTCAATGGGCAGAC
T-bet	GTCCAACAATGTGACCCAGAT	ACCTCAACGATATGCAGCCG
IL4	GAAATCAGGTGTCCCATTCCAG	TGGGGAGGTCGCTTCTCAA
GATA3	GCCCCTCATTAAGCCCAAG	TTGTGGTGGTCTGACAGTTCG
STAT3	ACCAGCAGTATAGCCGCTTC	GCCACAATCCGGGCAATCT
GAPDH	GGAGCGAGATCCCTCCAAAAT	GGCTGTTGTCATACTTCTCATGG

Supplemental Table 2. Antibody for IHC used

Antibody	Manufacturers	Dilution rate
HLA-DAP1	Proteintech	1:350
CIITA	Bioss	1:350

Supplemental Table 3. AEG-1 is associated with immune cells

**Clinicopathological Characteristics**	Overall survial (n=7462)			RFS (n=4420)		
	N	Harzard ratio	P-value	N	Harzard ratio	P-value
**Basophils**						
enriched	3319	1.79(1.16-2.76)	**0.0072**	3319	1.57(0.47-5.24)	0.46
decreased	3698	0.84(0.59-1.2)	0.34	3698	2.55(0.58-11.25)	0.2
**B-cells**						
enriched	3763	1.66(1.08-2.54)	**0.02**	3763	0.57(0.2-1.66)	0.3
decreased	3254	1.29(0.9-1.86)	0.17	3254	289579458.82(0-lnf)	0.052
CD4+ memory T-cells						
enriched	3791	1.59(1.11-2.28)	**0.01**	3791	1.85(0.63-5.48)	0.26
decreased	3226	0.75(0.49-1.17)	0.2	3226	1008122518.68(0-lnf)	0.098
CD8+ T-cells						
enriched	3601	1.41(0.97-2.05)	0.067	3601	0.64(0.28-1.48)	0.29
decreased	3416	1.6(1.02-2.5)	**0.039**	3416	1271481517.77(0-lnf)	0.052
Eosinophils						

enriched	3160	1.35(0.88-2.09)	0.17	3160	2.59(0.32-21.11)	0.36
decreased	3857	1.61(1.11-2.33)	**0.011**	3857	2.06(0.75-5.69)	0.15
Macrophages						
enriched	3983	1.56(1.05-2.33)	**0.028**	3983	1.88(0.59-6.03)	0.28
decreased	3034	1.27(0.88-1.82)	0.2	3034	7.4(0.94-58.03)	**0.028**
Mesenchymal stem cells						
enriched	3508	1.27(0.88-1.82)	0.2	3508	306431406.76(0-lnf)	**0.026**
decreased	3509	2.05(1.3-3.26)	**0.0018**	3509	0.45(0.14-1.43)	0.16
Natural killer T-cells						
enriched	3165	2.17(1.1-4.27)	**0.021**	3165	2.18(0.54-8.76)	0.26
decreased	3852	1.58(1.12-2.24)	**0.0086**	3852	2.03(0.67-6.16)	0.2
Regulatory T-cells						
enriched	3434	1.6(1.13-2.28)	**0.0081**	3434	2.67(0.78-9.09)	0.1
decreased	3583	1.56(1-2.43)	**0.049**	3583	290583980.78(0-lnf)	0.12
Type 1 T-helper cells						
enriched	3334	1.35(0.92-1.99)	0.13	3334	2.05(0.7-6.03)	0.18

decreased	3683	2.49(1.23-5.06)	**0.009**	3683	0-(0-lnf)	0.15
Type 2 T-helper cells						
enriched	3942	1.32(0.99-1.76)	0.061	3942	1.65(0.67-4.08)	0.27
decreased	3075	4.87(0.53-42.82)	0.12	3075	-	-
